# Lineage-Specific Expression of Bestrophin-2 and Bestrophin-4 in Human Intestinal Epithelial Cells 

**DOI:** 10.1371/journal.pone.0079693

**Published:** 2013-11-05

**Authors:** Go Ito, Ryuichi Okamoto, Tatsuro Murano, Hiromichi Shimizu, Satoru Fujii, Toru Nakata, Tomohiro Mizutani, Shiro Yui, Junko Akiyama-Morio, Yasuhiro Nemoto, Eriko Okada, Akihiro Araki, Kazuo Ohtsuka, Kiichiro Tsuchiya, Tetsuya Nakamura, Mamoru Watanabe

**Affiliations:** 1 Department of Gastroenterology and Hepatology, Graduate School, Tokyo Medical and Dental University, Tokyo, Japan; 2 Department of Advanced GI therapeutics, Graduate School, Tokyo Medical and Dental University, Tokyo, Japan; Université de Sherbrooke, Canada

## Abstract

Intestinal epithelial cells (IECs) regulate the absorption and secretion of anions, such as HCO3^-^ or Cl^-^. Bestrophin genes represent a newly identified group of calcium-activated Cl^-^ channels (CaCCs). Studies have suggested that, among the four human bestrophin-family genes, bestrophin-2 (BEST2) and bestrophin-4 (BEST4) might be expressed within the intestinal tissue. Consistently, a study showed that BEST2 is expressed by human colonic goblet cells. However, their precise expression pattern along the gastrointestinal tract, or the lineage specificity of the cells expressing these genes, remains largely unknown. Here, we show that BEST2 and BEST4 are expressed *in vivo*, each in a distinct, lineage-specific manner, in human IECs. While BEST2 was expressed exclusively in colonic goblet cells, BEST4 was expressed in the absorptive cells of both the small intestine and the colon. In addition, we found that BEST2 expression is significantly down-regulated in the active lesions of ulcerative colitis, where goblet cells were depleted, suggesting that BEST2 expression is restricted to goblet cells under both normal and pathologic conditions. Consistently, the induction of goblet cell differentiation by a Notch inhibitor, LY411575, significantly up-regulated the expression of not BEST4 but BEST2 in MUC2-positive HT-29 cells. Conversely, the induction of absorptive cell differentiation up-regulated the expression of BEST4 in villin-positive Caco-2 cells. In addition, we found that the up- or down-regulation of Notch activity leads to the preferential expression of either BEST4 or BEST2, respectively, in LS174T cells. These results collectively confirmed that BEST2 and BEST4 could be added to the lineage-specific genes of humans IECs due to their abilities to clearly identify goblet cells of colonic origin and a distinct subset of absorptive cells, respectively.

## Introduction

The intestinal mucosa plays an important role in both the absorption and secretion of water and electrolytes to maintain homeostasis in the human body. Ion channels create a trans-epithelial osmotic gradient, thereby regulating the passive movement of water between the mucosa and the intestinal lumen[1]. The secretion and absorption of Cl^-^ and HCO3^-^ plays a major role in this process, and thus excess secretions of Cl^-^ represent a common underlying mechanism of various diarrheal diseases, such as cholera [2,3].

Previous studies have suggested that at least two types of Cl^-^ channels are expressed on the apical side of human intestinal epithelial cells (IECs): cystic fibrosis conductance regulator (CFTR) [4] and calcium-activated Cl^-^ channels (CaCCs) [3,5]. These channels co-operate with the basolaterally located transporter, NKCC1, and play major roles in the secretion of Cl^-^ [1,3]. In addition, Cl^-^/HCO_3_^-^ transporters, such as DRA, are expressed at the apical membrane of human IECs [2,3,6], where their function is critically required for the electroneutral absorption of NaCl [1,4] and the secretion of HCO_3_^-^ [3,5,7]. Thus, the concerted function of these channels and transporters is indispensable to maintain intestinal fluid homeostasis. 

However, studies have further emphasized the importance of intestinal HCO_3_^-^ secretion in another aspect [8]. It has been suggested that the secretion of HCO3^-^ is indispensable for proper mucin release [9,10] and that the impairment of such function results in the mucus accumulation that is found in cystic fibrosis patients [11]. Thus, the trans-epithelial secretion of HCO3^-^ complements the function of mucin-producing goblet cells, thereby contributing to fully maintain the innate immune function of intestinal mucin. 

Bestrophins are newly identified genes encoding ion channels that can function as Cl^-^ channels, HCO3^-^ channels or voltage-gated Ca^2+^ channels [12]. So far, four members of this gene family (BEST1, BEST2, BEST3 and BEST4) have been identified in humans [13]. BEST1 was first identified as the gene responsible for vitelliform macular dystrophy, an ophthalmologic disease also known as the Best disease [14,15]. While human BEST1 appears to be highly expressed in the retina, studies have suggested that other members of this gene family, such as BEST2 and BEST4, may be predominantly expressed within the human colon [16]. A previous study by Yu et al showed that BEST2 is specifically expressed by goblet-shaped epithelial cells in the colons of both humans and mice [17]. In the mouse colon, these BEST2-positive cells were confirmed to be MUC-2-positive goblet cells. However, it remains uncertain whether both BEST2 and BEST4 are expressed by MUC-2 positive goblet cells in the human intestine.

 Additionally, in the previous study, it was shown that BEST2-null mice spontaneously develop mild colitis [17]. In addition, the study showed that the induction of colitis in those mice significantly exacerbated inflammation, delaying disease recovery and indicating that BEST2 may play a distinct role in both the initiation and the persistence of colitis. However, neither the expression of bestrophin genes in inflammatory bowel disease patients nor its relevance to disease activity has ever been described.

In this study, we show that human IECs express BEST2 and BEST4 at their basolateral membrane, each in a distinct lineage-specific manner. BEST2 appears to be a colonic goblet cell-specific gene, whereas BEST4 is an absorptive cell-specific gene that is found both in the small intestine and in the colon. Such a lineage-specific expression was conserved in pathological conditions, as BEST2 expression was markedly down-regulated in active lesions of ulcerative colitis (UC) patients, in whom goblet cells are depleted. In addition, a series of studies using *in vitro* differentiation models consistently confirmed the lineage-specific expressions of BEST2 and BEST4 in human IECs. Our present findings suggest that both BEST2 and BEST4 can be considered among the lineage-specific genes in the human intestine that clearly identify a colonic, but not small intestinal, origin of goblet cells and a subset of absorptive-lineage cells, respectively. 

## Materials and Methods

### Ethics statement

The study was approved by the institutional review board of Yokohama Municipal General Hospital and Tokyo Medical and Dental University. Written informed consent was obtained from all patients.

### Human intestinal tissue specimens

Human tissue specimens were obtained from patients who underwent surgery for the treatment of Crohn’s disease, ulcerative colitis or colon cancer at Yokohama Municipal General Hospital or Tokyo Medical and Dental University Hospital. Normal intestinal tissue was obtained from a non-inflamed region from the small intestines of Crohn’s disease patients or from non-tumorous regions of colon cancer patients. 

### Immunohistochemistry

Immunohistochemistry using human intestinal tissues has been described elsewhere [18-20]. The primary antibodies that were used in the present study were as follows: anti-bestrophin2 (1:500, PAB24487, Abnova, Taipei, Taiwan), anti-bestrophin4 (1:200, C-19, Santa-Cruz Biotechnology, Dallas, Texas, USA), anti-human MUC2 (1:100, Ccp58, Santa Cruz Biotechnology, Dallas, Texas, USA), anti-CD10 (1:80, Serotec, Raleigh, North Carolina, USA), anti-human E-Cadherin (1:1000, cloneHECD-1, Takara Bio, Ohtsu, Japan), anti-Hematopoietic prostaglandin D synthase (HPGDS) (1:1000, Cayman chemicals, Ann Arbor, Michigan, USA) and anti-Villin (1:100, Millipore, Billerica, Massachusetts, USA). Microwave treatment (500 W, 10 min) in 10 mM citrate buffer was required for staining BEST4, CD10, E-Cadherin, HPGDS and Villin. Primary antibodies were visualized by secondary antibodies conjugated with either Alexa-594 or Alexa-488 (Molecular Probes, Eugene, Oregon, USA). Goblet cell mucin was visualized by wheat-germ agglutinin (WGA) conjugated with Alexa-594 (1:100, Molecular Probes, Eugene, Oregon, USA).

### Cell culture

HT-29 cells and LS174T cells were purchased from ATCC (Manassas, Virginia, USA). Caco-2 cells were purchased from DS Pharma Biomedical (Osaka, Japan). Cells were maintained as described elsewhere [21,22]. The induction of goblet cell differentiation using HT-29 cells was performed by using LY411575 as previously described [21]. The induction of absorptive cell differentiation using Caco-2 cells was achieved by sustained culture under full confluency [23]. A cell line in which the expression of the intracellular domain of Notch1 (LS174T-NICD cells) can be induced under the control of doxycycline (DOX, 100 ng/ml, Clontech, Moutain View, California, USA) has been described elsewhere [24].

### siRNA-mediated gene knockdown

siRNA-mediated gene knockdowns were performed as previously described [25]. Briefly, a non-targeting control siRNA or a siRNA targeted to human MUC2 (100 nM Dharmacon, Lafayette, Colorado, USA), was transfected into cells using Lipofectamine RNAiMAX (Invitrogen, Carlsbad, California, USA) following the manufacturer’s instructions.

### Quantitative RT-PCR assays

Quantitative RT-PCR was performed as described elsewhere [21]. Briefly, 1 μL of each reverse-transcription product was subjected to a PCR reaction using SYBR green master mix (QIAGEN, Valencia, California, USA), which was run by ABI 7500 (Applied Biosystems, Foster City, California, USA). The primer sequences for human β-actin, Hes1, Sucrase-Isomaltase and Muc2 have been previously described [21,23]. The primer sequences for other genes were as follows: BEST2, 5´-ATG CGC GGT TGT CCC CGA AG-3´ (sense) and 5´-GTA AGC GGT TCG GGA CCC GC-3´ (antisense); BEST4, 5´-CAT GTA CGT GCC TCT CAC CA-3´ (sense) and 5´-TCT GTT CAG CCA CCT TGA GC-3´ (antisense); Villin, 5´-CCC TGG AGC AGC TAG TGA AC-3´ (sense) and 5´-GCT CAT AGG CAC ATC TGC AA-3´ (antisense); DPP-4, 5´-GAC GGA GTC CTG GGT TTC AG-3´ (sense) and 5´-CTC CAA CCT CAC GTG GAC AG-3´ (antisense). Each assay was performed in triplicate. The data were statistically analyzed with a paired Student’s t-test.

### Immunostaining of cultured cells

The staining of cultured cells was performed as previously described [24]. The primary antibodies used for staining BEST2, BEST4, MUC2 and Villin were the same as those used in immunohistochemistry. The other primary antibodies used were as follows: anti-Sucrase-Isomaltase (1:200, ATLAS antibodies, Stockholm, Sweden) and anti-cleaved Notch1 (Val1744) (1:100, Cell Signaling Technology, Danvers, Massachusetts). The detection of primary antibodies was carried out as described in the immunohistochemistry section. Data were collected using a conventional epifluorecent microscope (BZ2000, KEYENCE, Tokyo, Japan) or a confocal fluorescent microscope (FLUOVIEW FV10i, OLYMPUS, Tokyo, Japan).

### Immunoblot analysis

Cell lysates were prepared using a RIPA Buffer supplemented with a protease inhibitor cocktail (Roche, Basel, Switzerland) and benzonaze (Novagen, Madison, Wisconsin, USA). Lysates were subjected to 10% SDS-PAGE and transferred to PVDF membranes, as previously described [24]. The primary antibodies that were used in the present study were as follows: anti-cleaved Notch1 (Val1744) (1:1000, Cell Signaling Technology, Danvers, Massachusetts), anti-Hes1 (1:4000, a kind gift from Dr. T. Sudo, Toray Industry) [26], and anti-β-actin (1:1000, Sigma-Aldrich, Buchs, Switzerland). The blots were visualized using Luminata Forte Western HRP Substrate (Millipore, Billerica, Massachusetts, USA). 

## Results

### Both BEST2 and BEST4 are expressed in human IECs *in vivo*

**Figure 1:**
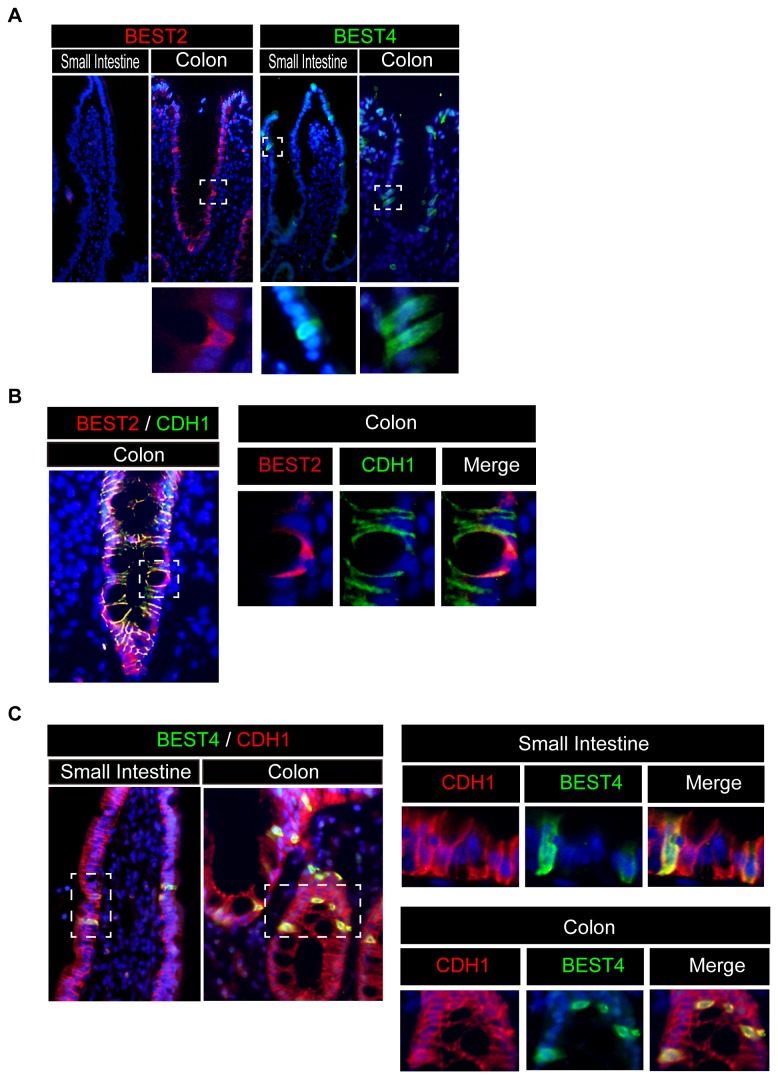
Human intestinal epithelial cells express both BEST2 and BEST4 *in* *vivo*. Representative data of three independent immunohistochemical analyses using human intestinal tissues are shown. (**A**) A single stain of BEST2 and BEST4 in the human small intestine and colon (Original magnification 200x). Goblet-shaped cells that are positive for BEST2 (red) are present in the colon but not in the small intestine. Columnar-shaped cells that are positive for BEST4 (green) are present both in the colon and in the small intestine. A magnified view of the squared area is shown in the lower column (Original magnification 400x). (**B**) Double-immunostaining of BEST2 with CDH1 (Original magnification 400x). In the human colon tissue, BEST2 (red) was expressed in CDH1-positive IECs (green). A magnified view of the squared area is shown in the right column (Original magnification 800x). (**C**) Double-immunostaining of BEST4 with CDH1 (Original magnification 400x). Both in the small intestine and in the colon, BEST4 (green) was expressed in CDH1-positive IECs (red). A magnified view of the squared area is shown in the right column (Original magnification 800x).

As former studies have suggested that the mRNA expression of BEST2 and BEST4 can be detected within the human intestinal tissue, we sought to determine the cell populations capable of expressing these ion channels. Consistent with previous research, immunohistochemical analysis revealed that BEST2-positive cells reside exclusively at the epithelial layer of the human colon [17]. Surprisingly, however, they were completely absent in the small intestine (Figure 1A). Such a colon-specific expression of BEST2 was confirmed in 3 individual subjects. In contrast, BEST4-positive cells were found at the epithelial layer of both small intestine and the colon (Figure 1A). All of the BEST2-positive IECs were distributed within the colonic crypt. BEST4-positive IECs were found at the small intestinal villus, colonic surface epithelium or at the upper part of the colonic crypt. The specificity of BEST2 and BEST4 staining was confirmed by comparing them to the staining using non-immune control IgG antibodies (Figure S1). Double-immunostaining analysis showed that all the BEST2- and BEST4-positive cells co-expressed CDH1 (Figure 1B, C), confirming that these cells were all IECs. Additionally, the subcellular distribution of both BEST2 and BEST4 mostly overlapped with that of CDH1, suggesting that these ion channels are predominantly located at the basolateral membrane of IECs (Figure 1B, C, right panel). These findings collectively confirmed that both BEST2 and BEST4 are expressed *in vivo* at the basolateral membrane of human IECs. 

### Human colonic goblet cells express BEST2 but do not express BEST4

**Figure 2:**
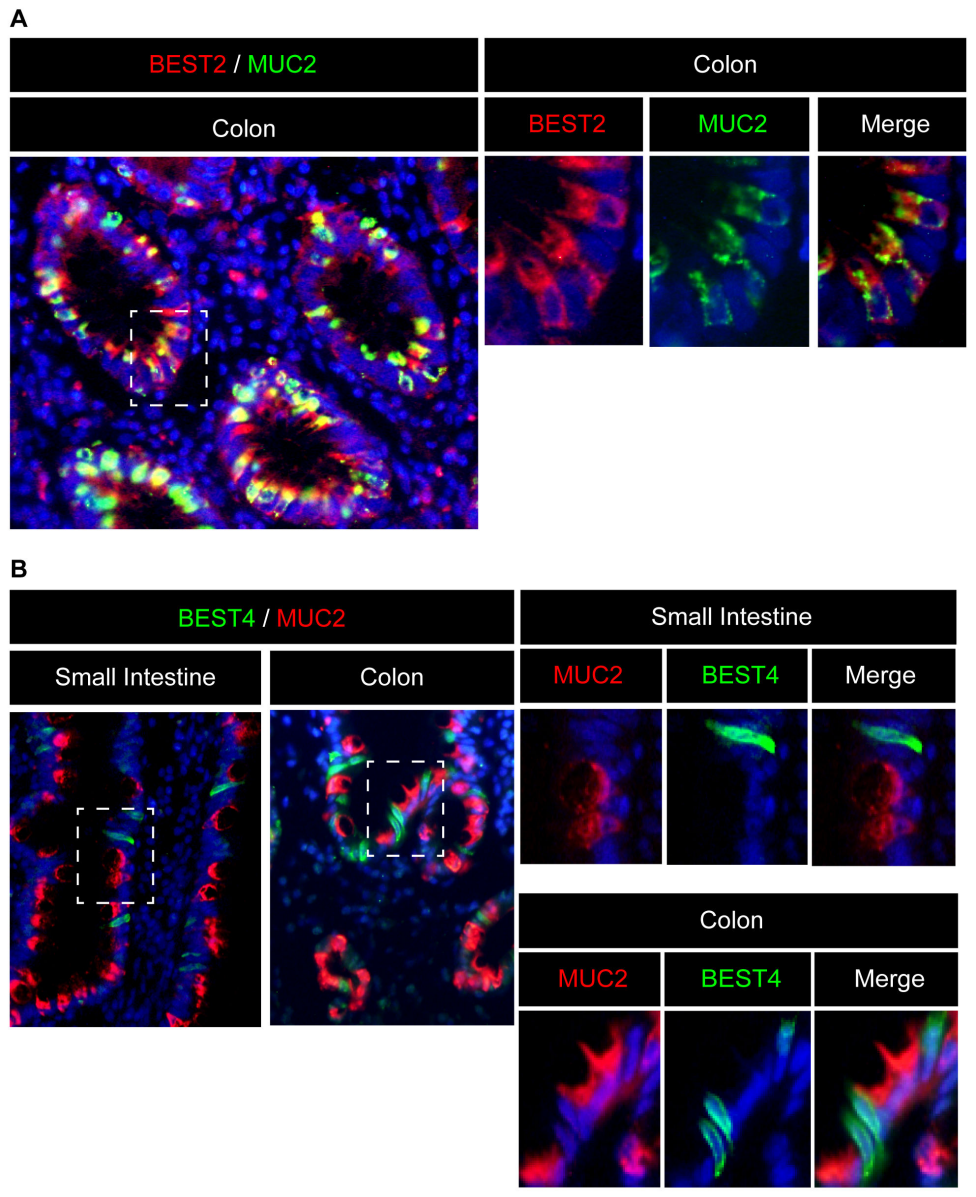
BEST2 is expressed specifically in human colonic goblet cells. (**A**) Double-immunostaining of BEST2 with MUC2 (Original magnification 400x). In the human colon tissue, BEST2 (red) was expressed exclusively in MUC2-positive IECs (green). A magnified view of the squared area is shown in the right column (Original magnification 800x). (**B**) Double-immunostaining of BEST4 with MUC2 (Original magnification 400x). Neither in the small intestine nor in the colon did BEST4-positive cells (green) co-express MUC2 (red). A magnified view of the squared area is shown in the right column (Original magnification 800x). Representative data of three independent analyses are shown.

Next, we tried to identify the lineages of IECs that are capable of expressing BEST2 and BEST4. Previous research has suggested that BEST2 is expressed in the goblet cells of mouse and human colons [17]. Our double-staining analysis confirmed that BEST2 is expressed exclusively by MUC2-positive IECs in the human colon (Figure 2A). However, in sharp contrast to BEST2, the co-staining of MUC2 and BEST4 showed that they are never co-expressed in either the small intestine or the colon (Figure 2B). This suggests that a distinct population of IECs other than goblet cells expresses BEST4. 

### Human intestinal absorptive cells express BEST4

**Figure 3:**
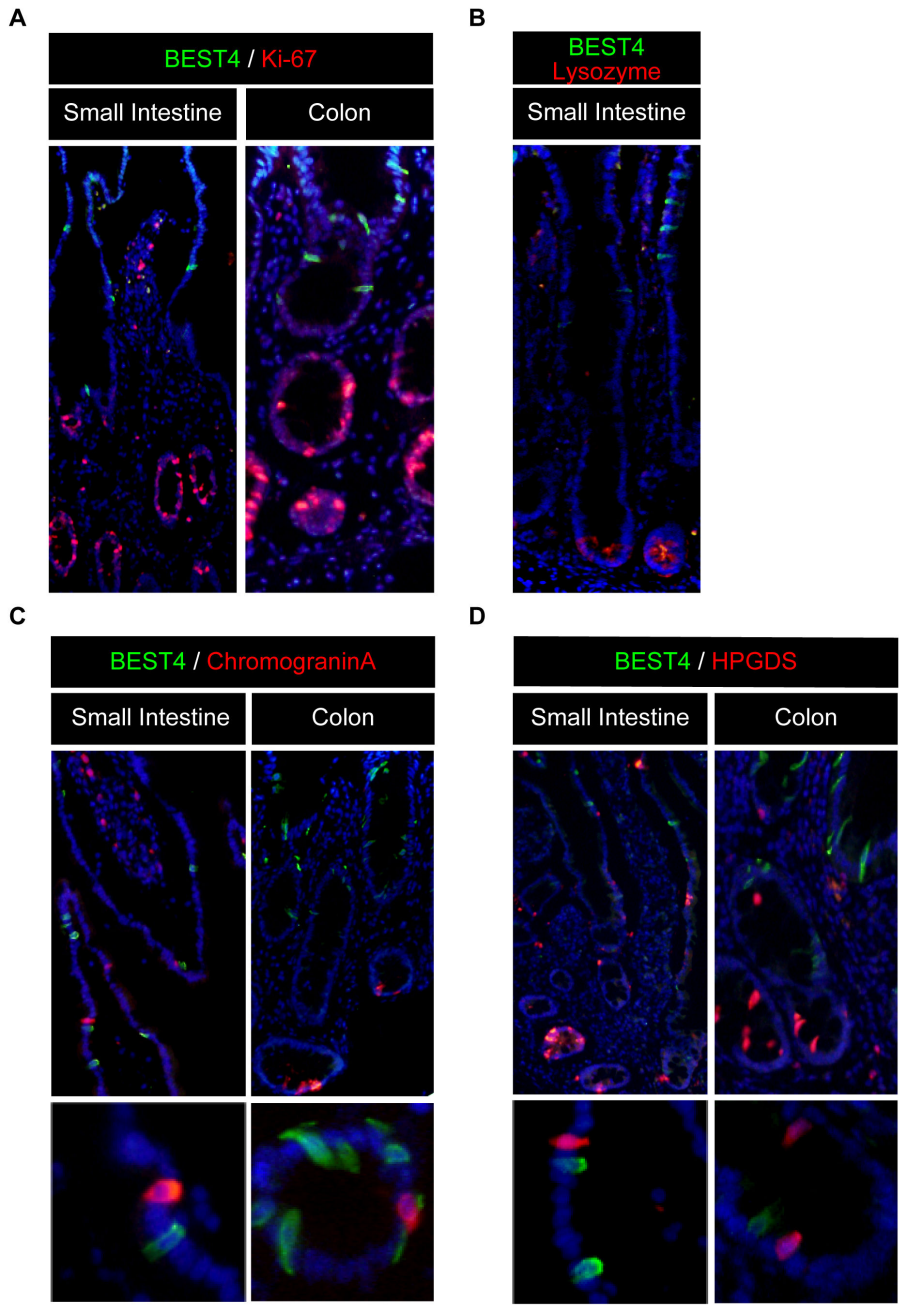
BEST4 is expressed in post-mitotic IECs other than secretory lineage cells. (**A**) Double-immunostaining of BEST4 with Ki-67 (Original magnification 200x). Neither in the small intestine nor in the colon did BEST4-positive cells (green) co-express Ki-67 (red). (**B**) Double-immunostaining of BEST4 with Lysozyme (Original magnification 200x). In the small intestine, BEST4-positive cells (green) did not co-express with Lysozyme (red). (**C**) Double immunostaining of BEST4 with Chromogranin-A (Original magnification 200x). Neither in the small intestine nor in the colon did BEST4-positive cells (green) co-express with Chromogranin-A (red). A magnified view is shown in the lower column (Original magnification 400x). (**D**) Double-immunostaining of BEST4 with HPGDS (Original magnification 200x). Neither in the small intestine nor in the colon did BEST4-positive cells (green) co-express with HPGDS (red). A magnified view is shown in the lower column (Original magnification 400x). Representative data of three independent analyses are shown.

We performed further analysis to identify the exact lineage of IECs that are capable of expressing BEST4. Co-staining with Ki-67 showed that BEST4-positive IECs are completely negative for this cell proliferation marker, suggesting that these cells are all post-mitotic both in the small intestine and in the colon (Figure 3A). In the small intestine, BEST4-positive IECs were clearly absent within the crypt region and never co-expressed lysozyme, a specific marker for Paneth cells (Figure 3B). In addition, BEST4-positive cells never co-expressed Chromogranin-A or HPGDS, indicating that BEST4 is expressed in cells other than enteroendocrine cells or tuft cells (Figure 3C, D). 

**Figure 4:**
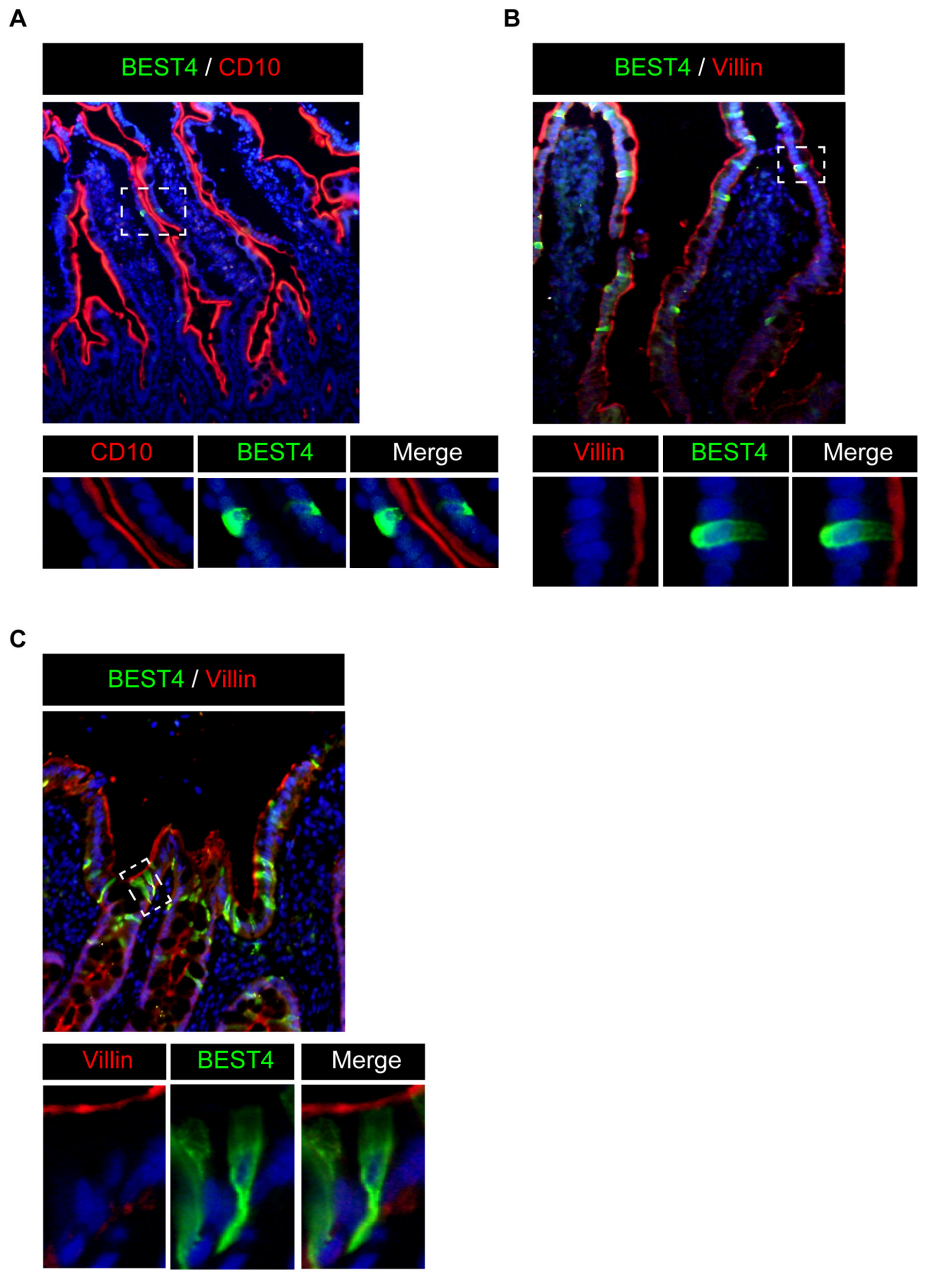
BEST4 is expressed in the absorptive cells of the human intestine *in* *vivo*. (**A**) Double-immunostaining of BEST4 with CD10 in the human small intestine (Original magnification 200x). In the human small intestinal tissue, BEST4 (green) was expressed in CD10-positive IECs (red). A magnified view of the squared area is shown in the lower column (Original magnification 400x). (**B**) Double-immunostaining of BEST4 with Villin in the human small intestine (Original magnification 400x). In the human small intestinal tissue, BEST4 (green) was expressed in Villin-positive IECs (red). A magnified view of the squared area is shown in the lower column (Original magnification 400x). (**C**) Double-immunostaining of BEST4 with Villin in the human colon (Original magnification 800x). In the colon, BEST4 (green) was expressed in Villin-positive IECs (red). A magnified view of the squared area is shown in the lower column (Original magnification 800x). Representative data of three independent analyses are shown.

We finally looked whether BEST4 is expressed in absorptive cells. Co-staining analysis of the small intestinal tissue showed that BEST4-positive IECs clearly co-express CD10 (Figure 4A). In addition, BEST4-positive IECs clearly co-expressed Villin at their apical side, both in the small intestine and in the colon (Figure 4B, C). These results showed that BEST4 is expressed specifically in human absorptive cells.

### Expression of BEST2 is markedly down-regulated in active lesions of UC

**Figure 5:**
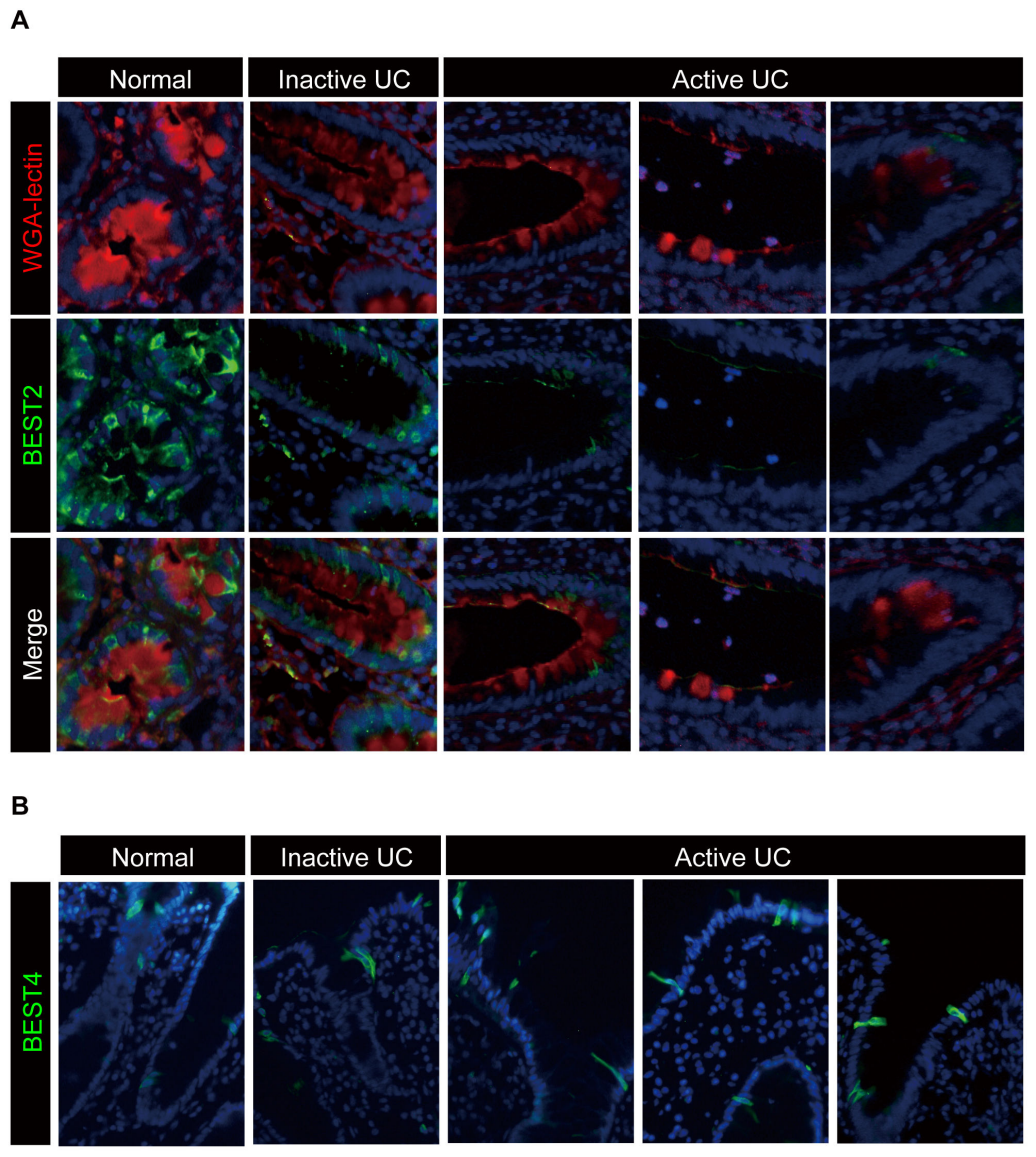
Expression of BEST2 is markedly down-regulated in active lesions of UC. The immunohistochemical analysis of BEST2 and BEST4 expression using colonic tissues of UC patients is shown. Representative data obtained from three subjects in each group are presented. (**A**) Expression of BEST2 is down-regulated in the active lesions of UC patients, where goblet cell mucins are depleted. The double staining of BEST2 (green) and WGA-lectin (red) using tissues from the normal human colon or from active and inactive lesions of UC patients is shown (Original magnification 400x). The red staining of WGA-lectin represents goblet cell mucins. Each series represents staining results obtained from a different patient (Original magnification 400x). (**B**) The expression of BEST4 is maintained at the surface epithelium of active, as well as inactive, lesions in UC patients. Single staining of BEST4 (green) using tissues from the normal human colon or from active and inactive lesions of UC patients is shown (Original magnification 400x). Each figure represents a staining result obtained from a different patient.

We next asked whether the lineage-specific expression of BEST2 or BEST4 is also maintained under pathological conditions. The depletion of goblet cells is one of the unique pathologic changes that is observed in active lesions of UC [27]. Therefore, we examined whether BEST2-positive cells are depleted in the active lesions of UC. The analysis of colonic tissues obtained from the three patients showed that BEST2 expression is markedly down-regulated in the active lesions of UC in which goblet cells are depleted (Figure 5A). In contrast, the expression of BEST4 was maintained at almost the same level at the colonic surface epithelium of UC patients, irrespective of disease activity (Figure 5B). These results suggest that the expression of BEST2 is strictly regulated to goblet cells in both normal and pathological conditions. In addition, such a dramatic down-regulation of BEST2 in the active lesions of UC also suggests the possible involvement of BEST2 depletion in the pathophysiology of the disease. 

### Induction of goblet cell differentiation *in vitro* promotes the expression of BEST2 in human IECs

**Figure 6:**
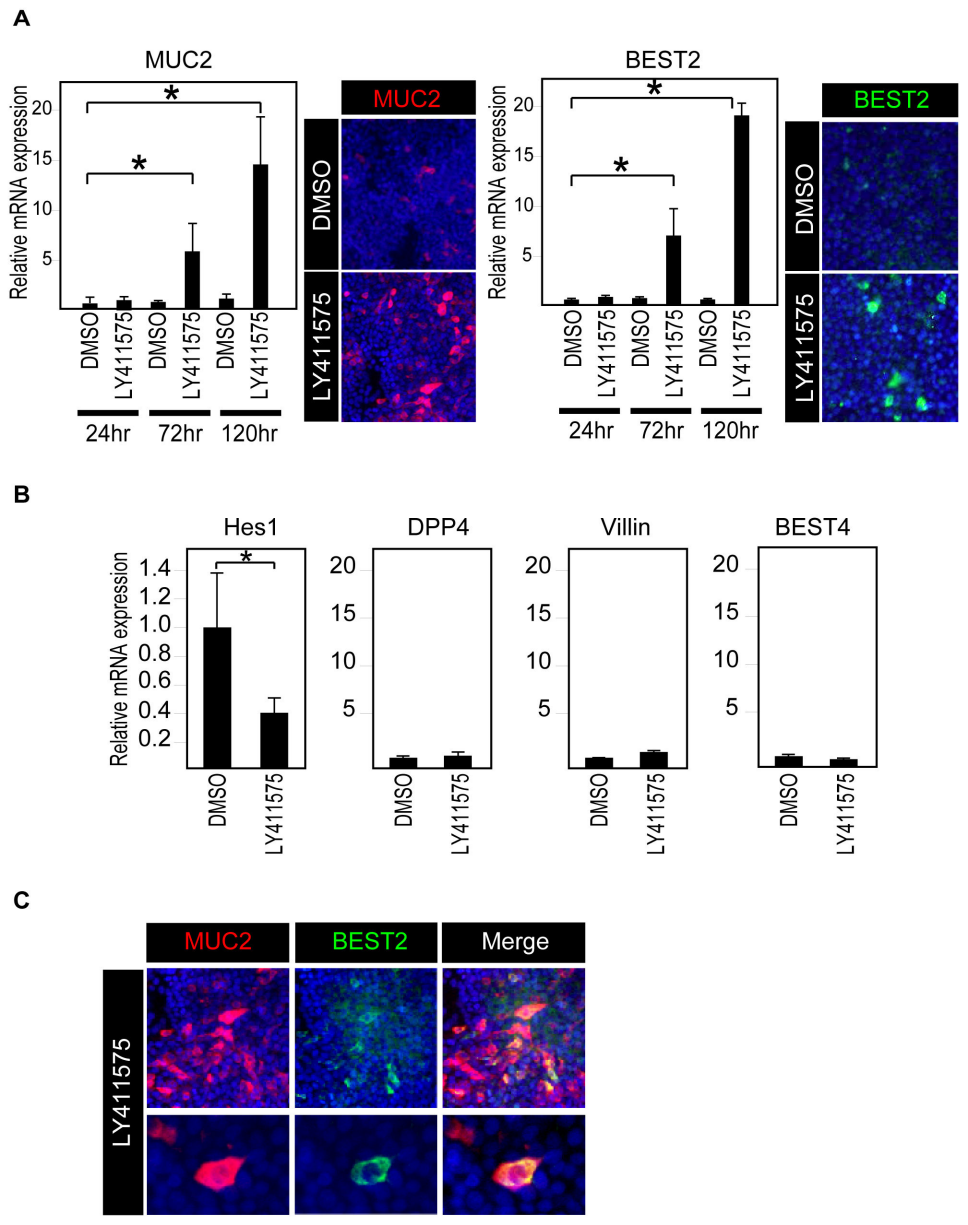
BEST2 expression is significantly up-regulated in HT-29 cells by the induction of goblet cell differentiation. (**A**) LY411575 induces the expression of BEST2 in HT-29 cells. The HT-29 cells were treated with either LY411575 (1μM) or DMSO for the indicated period of time and then subjected to quantitative RT-PCR analysis or immunostaining. The quantitative data were normalized to the expression levels of β-actin. Error bars represent the S.D. of triplicate experiments. * represents *P*< 0.05 compared to DMSO of the corresponding time period, as determined by Student’s *t*-test. The immunostaining of MUC2 (red, left panel) and BEST2 (green, left panel) after 120 h of treatment showed a clear increase in the number of MUC2- or BEST2- positive cells by LY411575 (Original magnification 200x). (**B**) The expression of absorptive cell marker genes, as well as BEST4, was not induced in HT-29 cells by LY411575. HT-29 cells were treated with either LY411575 or DMSO for 72 h and subjected for quantitative RT-PCR analysis. Data were normalized to expression levels of β-actin. Error bars represent S.D. of triplicate experiments. * represents *P*< 0.05 compared to DMSO, determined by Student’s *t*-test. (**C**) LY411575 induces BEST2 expression in MUC2-positive cells. HT-29 cells were treated with either LY411575 or DMSO for 120 h and subjected to double-immunostaining. The analysis of LY411575-treated cells revealed that BEST2-positive (green) cells clearly co-localize within MUC2-positive (red) cells (Original magnification 200x for upper series, 400x for lower series).

Our *in vivo* results suggested that the expression of BEST2 is restricted to goblet cells. However, whether the expression of BEST2 is a part of goblet cell differentiation or a phenotype that is acquired after the goblet cells have matured remains uncertain. Therefore, we used an *in vitro* goblet cell differentiation model to examine the expression levels of BEST2. In our previous study, we showed that a γ-secretase inhibitor, LY411575, can promote the goblet cell differentiation of HT-29 cells through the inhibition of Notch signaling [24]. Using the same model, we found that LY411575 can not only up-regulate the expression of MUC2 but can also up-regulate the expression of BEST2 at both the mRNA and protein levels (Figure 6A). Further analysis of the cells treated by LY411575 for 72 h confirmed that BEST2 expression is induced when Notch signaling has been down-regulated, as shown by the down-regulation of Hes1 mRNA expression (Figure 6B). In sharp contrast to BEST2 and MUC2, the mRNA expression of absorptive cell-specific genes, such as DPP4, Villin and BEST4, showed no significant change (Figure 6B). Double immunostaining confirmed that BEST2 expression is induced exclusively within MUC2-positive cells by the LY411575 treatment of HT-29 cells (Figure 6C). However, the concomitant expression of MUC2 is not necessarily required for BEST2 expression, as the siRNA-mediated knockdown of MUC2 had no effect on LY411575-induced BEST2 expression in HT-29 cells (Figure S2). These findings collectively show that BEST2 is one of the genes that is up-regulated during the process of human intestinal goblet cell differentiation. 

### Induction of absorptive cell differentiation *in vitro* promotes expression of BEST4 in human IECs

**Figure 7:**
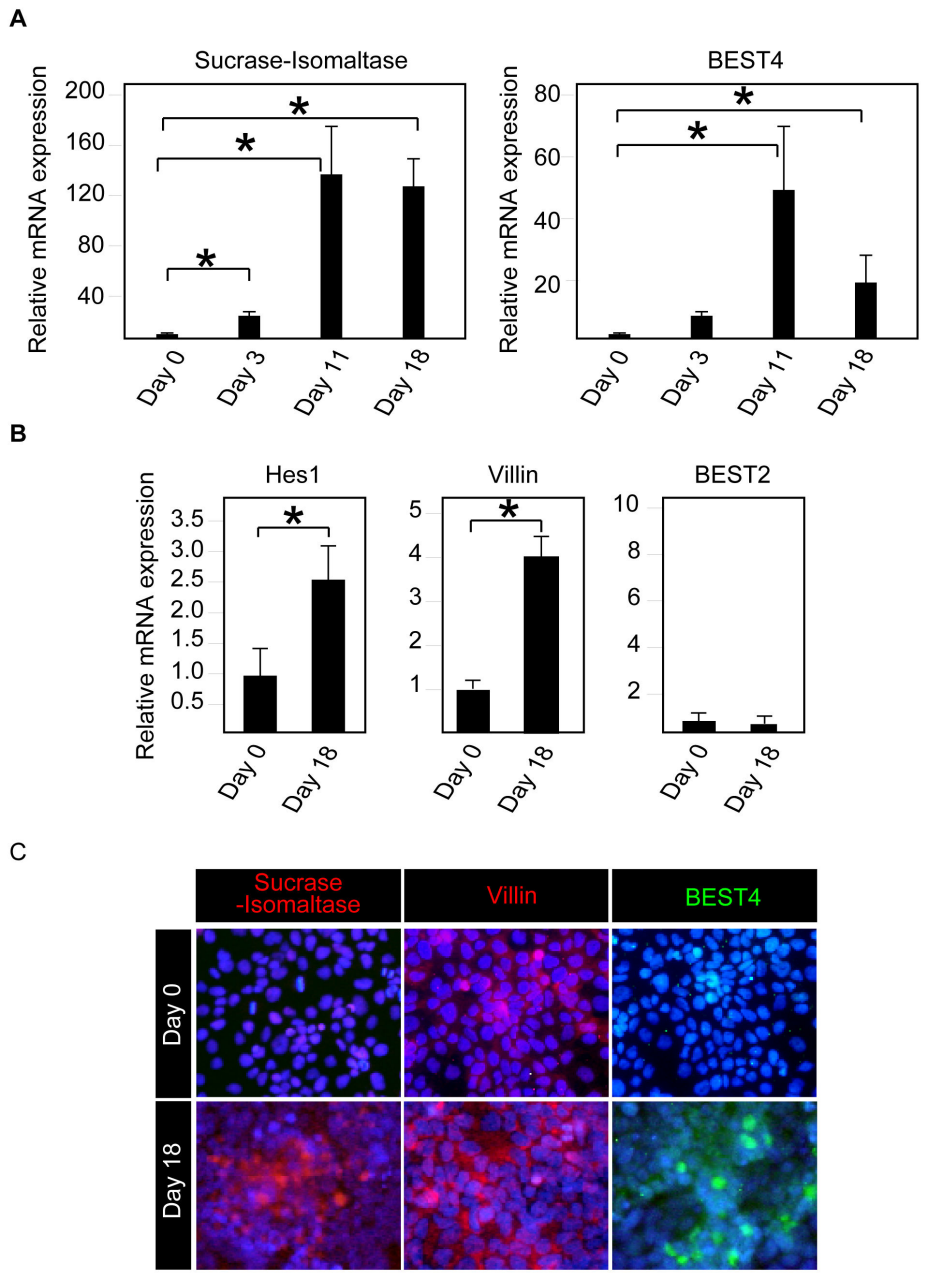
BEST4 expression is significantly up-regulated in Caco-2 cells by the induction of absorptive cell differentiation. (**A**) Induction of absorptive cell differentiation induces expression of both BEST4 and Sucrase-Isomaltase, in Caco-2 cells. Caco-2 cells were cultured under full confluency for the indicated period of time and subjected to quantitative RT-PCR analysis. The data were normalized to the expression levels of β-actin. Error bars represent the S.D. of triplicate experiments. * represents *P*< 0.05 compared to day 0, as determined by Student’s *t*-test. (**B**) The expression of not BEST2, but of absorptive cell marker genes, was induced in Caco-2 cells by the induction of absorptive cell differentiation. Caco-2 cells were cultured in full confluency for 18 days and subjected to quantitative RT-PCR analysis. The data were normalized to the expression levels of β-actin. Error bars represent the S.D. of triplicate experiments. * represents *P*< 0.05 compared to day 0, as determined by Student’s *t*-test. (**C**) The induction of absorptive cell differentiation generated BEST4 expression in Villin- or Sucrase-Isomaltase- positive cells. Caco-2 cells were cultured in full confluency for 18 days and subjected to immunocytochemistry. Staining of Sucrase-Isomaltase, Villin and BEST4 showed clear increases in the number of BEST4-positive cells (green) at day 18 within the area where the expression of Sucrase-Isomaltase (red) or Villin (red) was uniformly induced (Original magnification 200x). Representative data of three independent analyses are shown.

Our *in vivo* analysis also showed that BEST4 is exclusively expressed by absorptive cells. Thus, we examined whether BEST4 expression is induced during the process of absorptive cell differentiation *in vitro*. A long-term culture of Caco-2 cells under full confluency is commonly used as an *in vitro* model of absorptive cell differentiation [23]. Maintaining Caco-2 cells at full confluency for up to 18 days clearly induced the mRNA expression of not only Sucrase-Isomaltase but also BEST4 (Figure 7A). The further analysis of the cells at day 18 showed that the mRNA expression of Hes1, as well as Villin, is also significantly up-regulated, indicating a concomitant activation of Notch signaling (Figure 7B). In sharp contrast, the mRNA expression of BEST2 remained completely unchanged (Figure 7B). The immunocytochemistry of differentiated Caco-2 cells confirmed that the expression of the BEST4 protein is induced within Villin-positive or Sucrase-Isomaltase-positive cells (Figure 7C). These results collectively showed that BEST4 expression is induced during the process of human absorptive cell differentiation. 

### Activation level of Notch signaling can alternatively potentiate expression of BEST2 or BEST4 in human IECs

**Figure 8:**
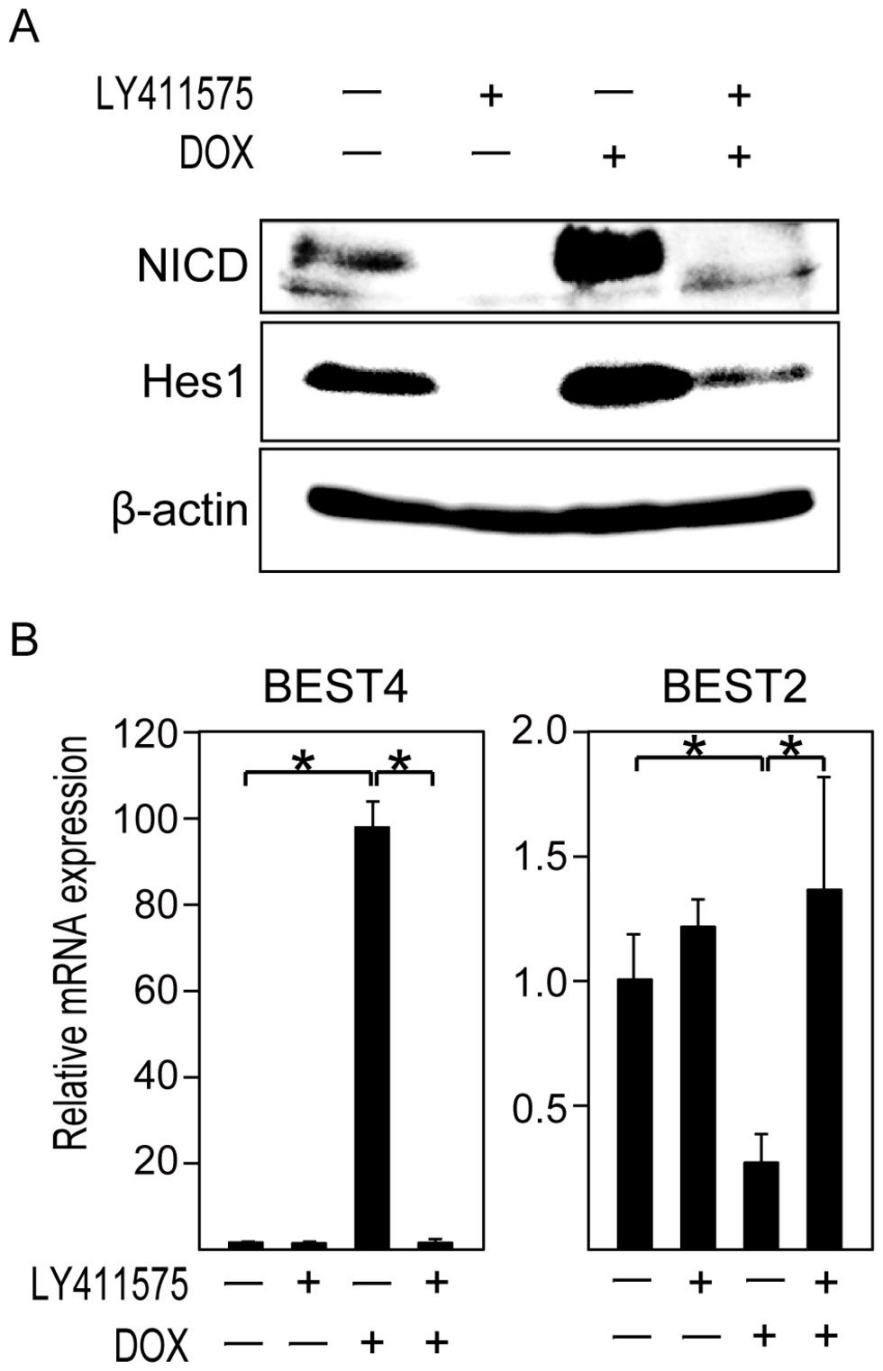
Activation level of Notch signaling can alternatively potentiate the expression of BEST2 or BEST4 in LS174T cells. An analysis of LS174T-NICD cells, a sub-line of LS174T cells in which we can induce expression of activated form of Notch1 (NICD) by doxycycline (DOX) addition, is shown. (**A**) The up- and down-regulation of the Notch activity level can be achieved by addition of DOX or LY411575 to LS174T-NICD cells. LS174T-NICD cells were pre-cultured with LY411575 (1μM) or DMSO for 48 h and then cultured with LY411575, DOX (100 ng/ml) or both for the following 24 h. Those cells were subjected to immunoblot analysis for NICD, Hes1 and β-actin. A membrane was stripped and re-probed with series of antibodies. (**B**) The activation level of Notch signaling may potentiate the expression of BEST2 and BEST4 in LS174T cells. LS174T-NICD cells were cultured as described in (A) and subjected to quantitative RT-PCR analysis. Data were normalized to the expression levels of β-actin. Error bars represent the S.D. of triplicate experiments. * represents *P*< 0.05, determined by Student’s *t*-test.

The results of our *in vitro* analysis using two different differentiation models indicate that the activation level of Notch signaling may not only determine the cell lineage decision [28] but may also provide a cellular context that favors the expression of either BEST2 or BEST4 in human IECs. To test such a hypothesis, we used a previously established LS174T-derived cell line, LS174T-NICD, in which we could force the expression of the Notch intracellular domain (NICD) in a doxycycline (DOX)-dependent manner [21,24,29]. The addition of DOX clearly up-regulated the expression of both NICD and Hes1 in LS174T-NICD cells, suggesting a high level of Notch activation (Figure 8A, lane 3). Conversely, treatment by LY411575 markedly down-regulated the expression of both NICD and Hes1 in LS174T-NICD cells, irrespective of the DOX addition, suggesting a minimal level of Notch activation (Figure 8A, lane 2 and 4). Thus, by combining DOX and LY411575 treatment, we were able to up- or down-regulate the activation level of Notch signaling in LS174T-NICD cells. 

Next, we examined whether the expression level of BEST2 and BEST4 is dependent on the activation level of Notch signaling in LS174T-NICD cells. Treatment by DOX alone significantly up-regulated the mRNA expression of BEST4 up to 100-fold in LS174T-NICD cells (Figure 8B). Such a dramatic increase of BEST4 expression by the over-expression of NICD was also confirmed at the protein level (Figure S3). In contrast, the expression of BEST2 was significantly down-regulated by the same treatment (Figure 8B). Although treatment by LY411575 alone showed only a minimal effect upon BEST2 and BEST4 expression in the present cell line, the response to DOX was completely abolished by adding both DOX and LY411575, resulting in a dramatic recovery of BEST2 expression and, conversely, a suppression of BEST4 expression (Figure 8B). From these results, we suggest that the activation level of Notch signaling may provide a cellular context that can alternatively potentiate the expression of BEST2 or BEST4 in human IECs. However, as the major population of Notch-activated cells *in vivo* is mitotic and resides at the crypt region [18], it is less likely that these genes are under the direct control of the Notch pathway. Alternatively, the activation level of Notch signaling during the progenitor cell period may help to establish a lineage-specific cell context that allows subsequent expression of either BEST2 or BEST4 *in vivo*. 

## Discussion

In this study, we found that human BEST2 is expressed exclusively in colonic goblet cells but is completely absent in small intestinal goblet cells. Several genes, including MUC2, have been reported to be expressed specifically in intestinal goblet cells [30,31]. However, those genes are expressed both in the small intestine and in the colon, suggesting that they represent genes and functions that are shared among goblet cells throughout the gastrointestinal tract. In contrast, the present expression pattern of BEST2 clearly indicates that the goblet cells in the human small intestine and in the colon have, at least in part, distinct gene expression profiles. The present results also suggest that a dysfunction in such a colonic goblet cell-specific gene might be involved in colon-specific gastrointestinal diseases, such as UC. 

We also found that BEST4 is expressed in a distinct subpopulation of absorptive cells. So far, no gene has been established as a marker to sub-classify absorptive-type cells. Our findings suggest that these absorptive-type cells might be divided into at least two subtypes, which can be identified as BEST4-positive or -negative cells. These two populations may play distinct roles in transepithelial HCO_3_^-^ and/or Cl^-^ exchanges at the intestinal surface epithelium. However, the functional differences between the BEST4-positive and -negative absorptive cells remain to be elucidated.

Our present study showed that the expression of BEST2 is markedly down-regulated in active lesions of UC, where goblet cells are depleted. We also showed that the expression of BEST2, as well as of BEST4, is affected by the intracellular activity of Notch signaling. Our previous studies have shown that, in the inflamed mucosa of UC, Notch signaling is highly activated in an increased number of IECs, thereby mediating goblet cell depletion [24,29]. Thus, our present results further highlight the role of Notch signaling in UC, where it may also mediate down-regulation of BEST2. Further, the expression level of BEST2 appeared to be inversely correlated with disease activity, indicating that it may be used as one of the disease activity markers of UC. Former studies have shown that the expression of ion transporters, such as NHE3 and DRA, is significantly down-regulated in active lesions of UC [32]; this mechanism was proposed as one of the molecular bases of severe diarrhea [33]. Our study adds to this conception, as the depletion of BEST2 may also play a role in the impaired water and ion movement in UC. Additionally, as BEST2-knockout mice spontaneously develop colitis, it remains possible that BEST2 may play some role in the inflammation of UC, as suggested with other ion transporters, such as NHE1 and NHE3 [34,35].

In contrast to BEST2, the expression of BEST4 appeared to be maintained by the surface IECs remaining at the active lesions of UC. Such an expression may help to compensate for the loss of BEST2 function in crypt IECs of the inflamed mucosa. 

 In summary, we showed in this study that two bestrophin genes, BEST2 and BEST4, are expressed in distinct cell lineages of human IECs. The expression analysis of these genes, in addition to that of a formerly identified set of lineage-specific genes, may help to further identify the differentiation status of human IECs towards goblet cells or absorptive cells. 

## Supporting Information

Figure S1 **Specificity of immunostaining using anti-BEST2 and anti-BEST4 antibodies.** (**A**)The specificity of the rabbit-anti-BEST2 antibody (Anti-BEST2 Ab, Abnova, PAB24487) was confirmed by comparing it to the control staining using non-immune rabbit IgG as a primary antibody. Primary antibodies were detected by Alexa 594-conjugated secondary antibody (Red). (**B**) The specificity of goat-anti-BEST4 antibody (Anti-BEST4 Ab, Santa Cruz Biotechnology, C-19) was confirmed by comparing it to the control staining using non-immune goat IgG as a primary antibody. Positive signaling was detected by Alexa 488-conjugated secondary antibody (Green).
(TIFF)

Figure S2 **LY411575-mediated induction of BEST2 expression is independent from MUC2 expression in HT-29 cells.** (**A**) The successful achievement of a siRNA-mediated knockdown of MUC2 in HT-29 cells. The cells were transfected with siRNA targeted to MUC2 (MUC2 siRNA) or with non-targeting control siRNA (Control siRNA) and then subjected to quantitative RT-PCR analysis after treatment with DMSO or LY411575 for 72 h. The results showed a 35% reduction of MUC2 mRNA expression in DMSO-treated cells and a 58% reduction in LY411575-treated cells by MUC2 siRNA, compared to control siRNA. * represents P<0.05, determined by Student’s t-test. (**B**) No significant effect was observed upon BEST2 induction by MUC2 gene knockdowns in HT-29 cells. The cells shown in (A) were further analyzed for BEST2 mRNA expression by quantitative RT-PCR. N.S. represents not significant at the 0.05 probability level, determined by Student’s t-test.
(TIF)

Figure S3 **Forced activation of Notch signaling promotes membrane-bound BEST4 expression in LS174T-NICD cells.** (**A**) LS174T-NICD cells were cultured with or without DOX (100 ng/ml) for 48 h, and subjected to immunocytochemistry. Single staining of BEST4 (green) and NICD (red) showed clear increase in the number of NICD- or BEST4- positive cells by addition of DOX (Original magnification 400x). (**B**) The membrane-bound BEST4 expression is induced in Notch activated LS174T-NICD cells. LS174T-NICD cells were cultured with DOX (100 ng/ml) for 48 h, and subjected to a double immunostaining of BEST4 (red) and NICD (green). The staining shows clear distribution of BEST4 (red) at the membrane of NICD (green) positive LS174T-NICD cells, as shown by the confocal view of the cell. The Z-stack images obtained from a series of confocal images are shown (Original magnification 300x).
(TIF)
