# Supplementary figures and images for: Lineage-Specific Expression of Bestrophin-2 and Bestrophin-4 in Human Intestinal Epithelial Cells
Source: PLoS One. 2013 Nov 5;8(11):e79693. doi: 10.1371/journal.pone.0079693 (PMC3818177; doi:10.1371/journal.pone.0079693)

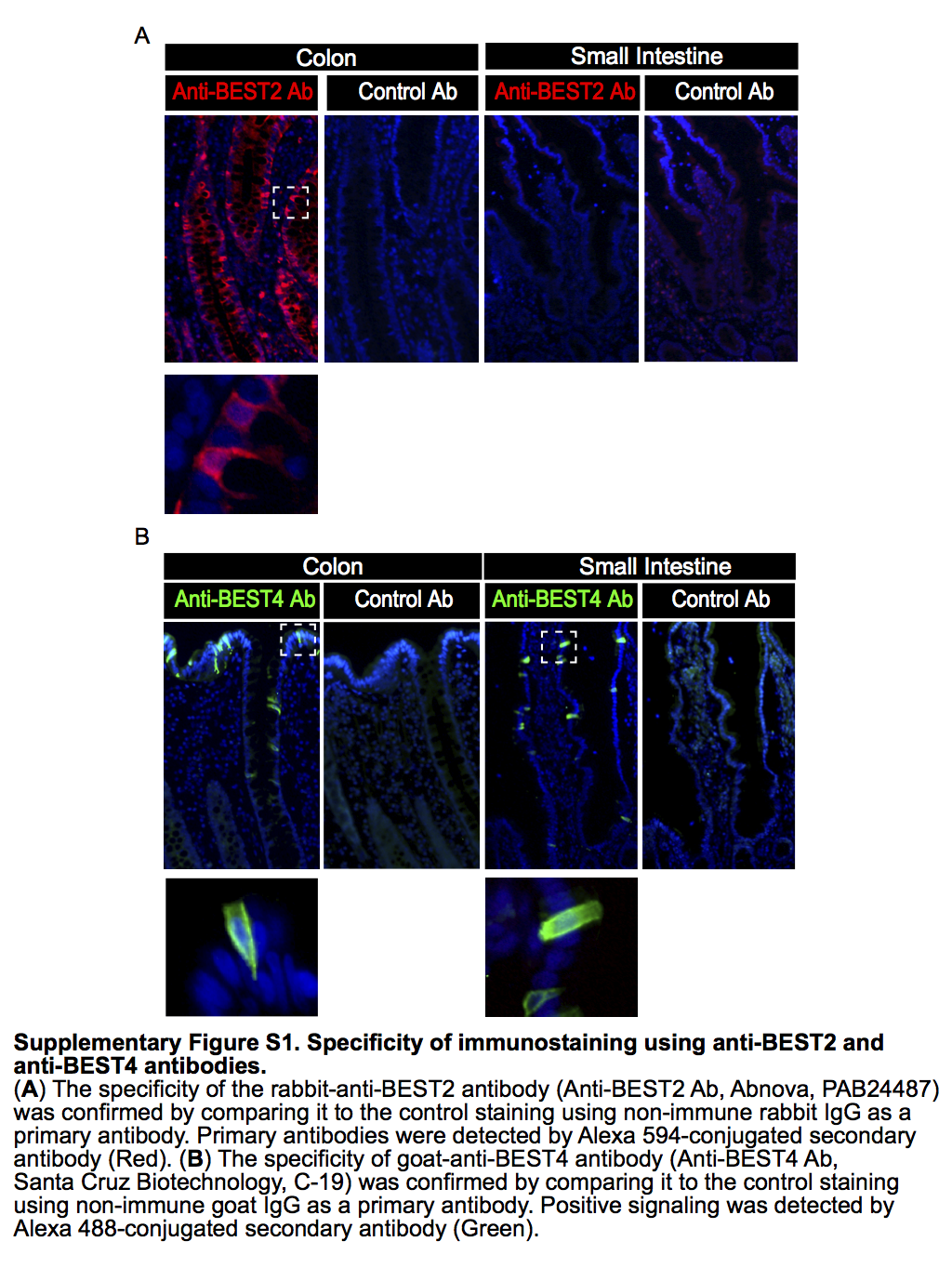

Supplement: Figure S1 — Specificity of immunostaining using anti-BEST2 and anti-BEST4 antibodies. (A)The specificity of the rabbit-anti-BEST2 antibody (Anti-BEST2 Ab, Abnova, PAB24487) was confirmed by comparing it to the control staining using non-immune rabbit IgG as a primary antibody. Primary antibodies were detected by Alexa 594-conjugated secondary antibody (Red). (B) The specificity of goat-anti-BEST4 antibody (Anti-BEST4 Ab, Santa Cruz Biotechnology, C-19) was confirmed by comparing it to the control staining using non-immune goat IgG as a primary antibody. Positive signaling was detected by Alexa 488-conjugated secondary antibody (Green). (TIFF) [file pone.0079693.s001.tiff]

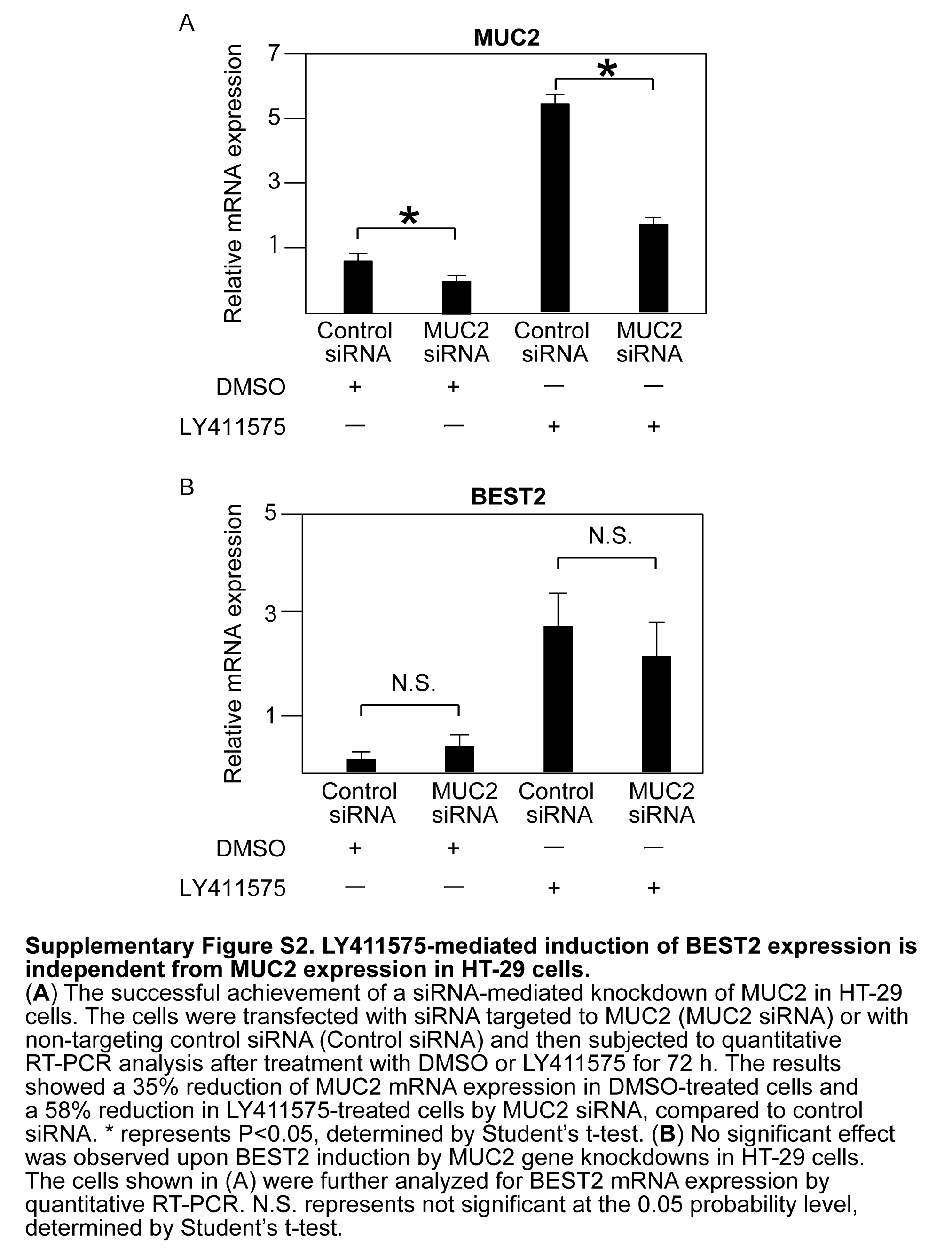

Supplement: Figure S2 — LY411575-mediated induction of BEST2 expression is independent from MUC2 expression in HT-29 cells. (A) The successful achievement of a siRNA-mediated knockdown of MUC2 in HT-29 cells. The cells were transfected with siRNA targeted to MUC2 (MUC2 siRNA) or with non-targeting control siRNA (Control siRNA) and then subjected to quantitative RT-PCR analysis after treatment with DMSO or LY411575 for 72 h. The results showed a 35% reduction of MUC2 mRNA expression in DMSO-treated cells and a 58% reduction in LY411575-treated cells by MUC2 siRNA, compared to control siRNA. * represents P<0.05, determined by Student’s t-test. (B) No significant effect was observed upon BEST2 induction by MUC2 gene knockdowns in HT-29 cells. The cells shown in (A) were further analyzed for BEST2 mRNA expression by quantitative RT-PCR. N.S. represents not significant at the 0.05 probability level, determined by Student’s t-test. (TIF) [file pone.0079693.s002.tif]

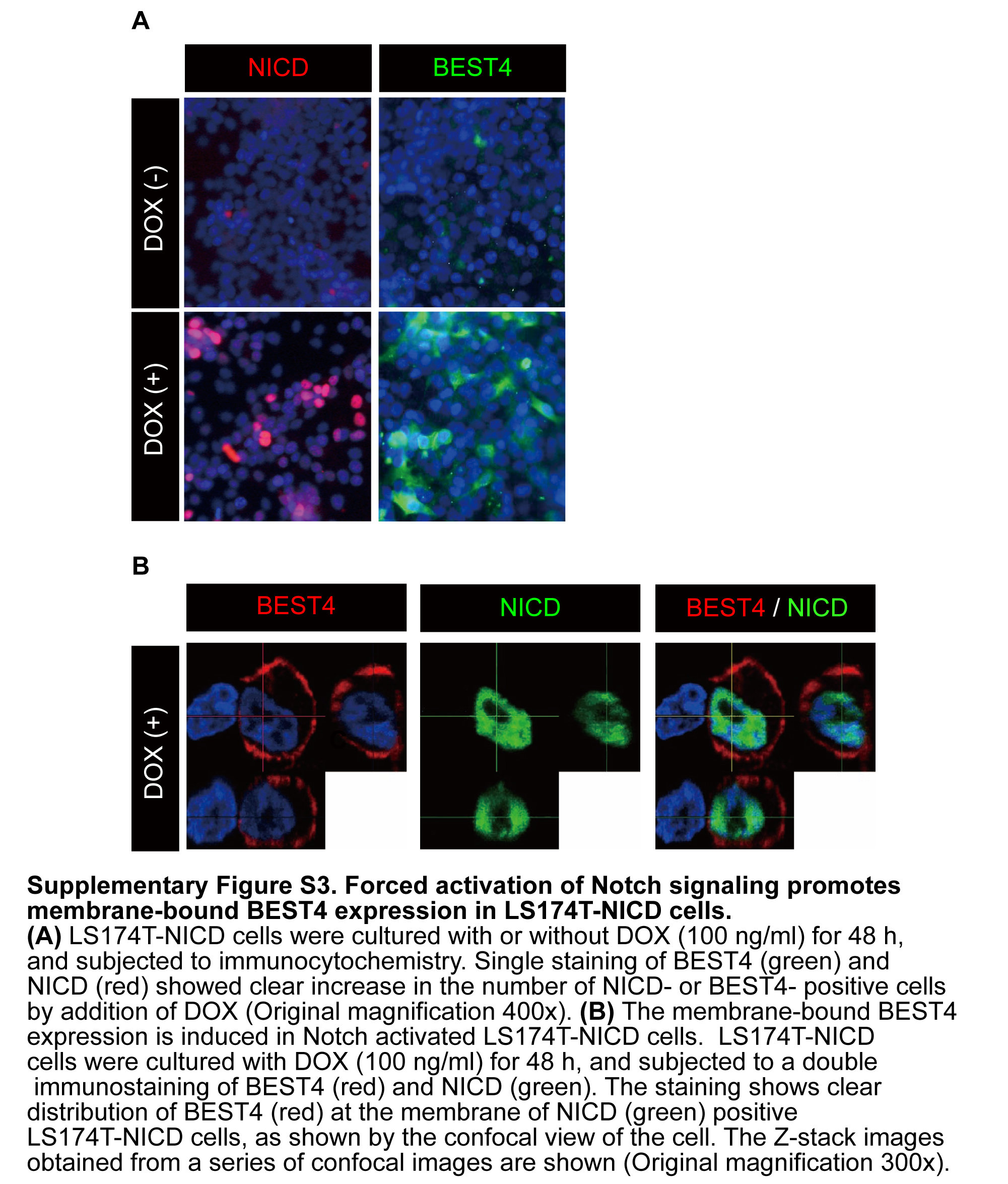

Supplement: Figure S3 — Forced activation of Notch signaling promotes membrane-bound BEST4 expression in LS174T-NICD cells. (A) LS174T-NICD cells were cultured with or without DOX (100 ng/ml) for 48 h, and subjected to immunocytochemistry. Single staining of BEST4 (green) and NICD (red) showed clear increase in the number of NICD- or BEST4- positive cells by addition of DOX (Original magnification 400x). (B) The membrane-bound BEST4 expression is induced in Notch activated LS174T-NICD cells. LS174T-NICD cells were cultured with DOX (100 ng/ml) for 48 h, and subjected to a double immunostaining of BEST4 (red) and NICD (green). The staining shows clear distribution of BEST4 (red) at the membrane of NICD (green) positive LS174T-NICD cells, as shown by the confocal view of the cell. The Z-stack images obtained from a series of confocal images are shown (Original magnification 300x). (TIF) [file pone.0079693.s003.tif]
